# Supplementary material for: Comprehensive Serum Glycopeptide Spectra Analysis (CSGSA): A Potential New Tool for Early Detection of Ovarian Cancer
Source: Cancers (Basel). 2019 Apr 27;11(5):591. doi: 10.3390/cancers11050591 (PMC6563019; doi:10.3390/cancers11050591)
Supplement: Supplementary file 1 [file cancers-11-00591-s001.pdf]

Supplementary Materials

Comprehensive Serum Glycopeptide Spectra Analysis (CSGSA): A Potential New Tool for Early Detection of Ovarian Cancer

Masaru Hayashi, Koji Matsuo, Kazuhiro Tanabe, Masae Ikeda, Mariko Miyazawa, Miwa Yasaka, Hiroko Machida, Masako Shida, Tadashi Imanishi, Brendan H. Grubbs, Takeshi Hirasawa and Mikio Mikami

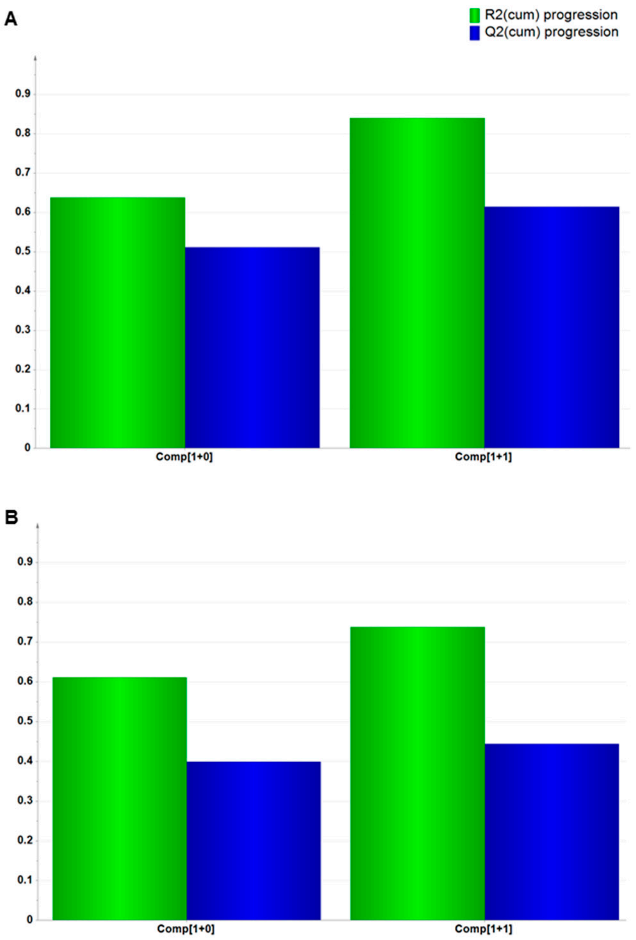

Figure S1. Validation sets for OPLS-DA. Model fit plots represent the results of cumulative percentages of variation of the data set (R2) and predictive values (Q2) using cross validation for first and second OPLS-DA components. (**A**) showed the study of early-stage EOC versus non-cancer, and (**B**) represents the study of early-stage OCCC versus endometrioma.

Table S1. Patient demographics.

Characteristic	Number (%)	Age
Benign disease	*n* = 45 (100%)	
Endometrioma	18 (40.0%)	43.78 (±5.48)
Ovarian cyst	17 (37.8%)	54.41 (±13.4)
Leiomyoma	10 (22.2%)	51.0 (±10.1)
Ovarian cancer	*n* = 39 (100%)	53.72 (±10.3)
Histology type		
Clear cell	15 (38.5%)	52.47 (±11.4)
Endometrioid	14 (35.8%)	53.71 (±10.3)
Serous	8 (20.5%)	54.13 (±9.8)
Mucinous	2 (5.1%)	61.50 (±6.3)
Stage		
I	21 (53.8%)	
II	2 (5.1%)	
III	15 (38.5%)	
IV	1 (2.6%)	
Histology-specific stage		
Clear cell		
I–II	9 (60.0%)	
III–IV	6 (40.0%)	
Endometrioid		
I–II	10 (71.4%)	
III–IV	4 (28.6%)	
Serous		
I–II	3 (37.5%)	
III–IV	5 (62.5%)	
Mucinous		
I–II	1 (50%)	
III–IV	1 (50%)	

Table S2. Exclusion criteria.

A history of hormonal drug administration due to malignant tumor, autoimmune disease, and thyroid abnormality
Affected by combination of several types of cancers
Abnormal values in blood tests. WBC: >9600, PLT: >48, LDH: >263, HB: <9.2 and CRP >3.0
Renal dysfunction
Liver dysfunction
Aged 75 and above
Diagnosed as mixed carcinoma
Diagnosed as fallopian tube cancer/peritoneal cancer
Affected massive ascites/pleural effusion
Diagnosed as mucinous cystoadenoma, germ cell tumor or sex cord stromal tumor

Supplementary Method S1

Serum Preparation

For this study, 10 μL of a 2 mg/mL aqueous solution of fetal calf fetuin (Sigma, St. Louis, MO, USA) was added to each serum sample (20 μL) to check the efficiency of trypsin digestion or the recovery of glycopeptides. Subsequently, trichloroacetic acid in acetone (100 mg/mL, 120 μL; Wako Pure Chemical Industries, Ltd, Osaka, Japan) was added to remove serum albumin. After mixing and centrifuging at 13,500× *g* for 5 min, the supernatant was removed, and cooled acetone (400 μL) was added to remove excess trichloroacetic acid. The suspension was centrifuged again at 13,500× *g* for 5 min, and the supernatant was removed. Finally, the precipitate was mixed with denaturing solution containing urea (80 μg; Wako Pure Chemical Industries), Tris-HCl buffer (pH 8.5, 100 μL), 0.1 M

EDTA solution (10 μL), 1 M Tris (2-carboxyethyl) phosphine hydrochloride (5 μL; Sigma) solution, and water (38 μL), and proteins were denatured for 10 min at 37 °C. Next, 1 M 2-iodoacetamide (40 μL; Wako Pure Chemical Industries) solution was added to the denaturing solution to protect the thiol residues in proteins. The solution was kept for 10 min at 37 °C in the dark, subsequently transferred into a 30 K ultrafiltration tube (Amicon Ultra 0.5 mL; Millipore Corp., Billerica, MA, USA) and centrifuged at 13,500× *g* for 30 min to remove denaturing reagents. The denatured proteins trapped on the filter were washed with 0.1 M Tris-HCl buffer (pH 8.5, 400 μL), followed by centrifugation at 13,500× *g* for 40 min. Next, 0.1 M Tris-HCl buffer (pH 8.5, 200 μL), 0.1 μg/μL trypsin (20 μL; Wako Pure Chemical Industries) solution, and 0.1 μg/μL lysyl endopeptidase (20 μL; Wako Pure Chemical Industries) solution were added to the ultrafiltration tube, and the denatured proteins on the filters were digested for 16 h at 37 °C. After digestion, the solution was centrifuged for 30 min at 13,500× *g*. The filtered solution, which contained digested peptides (including glycopeptides), was transferred to a 10 K ultrafiltration tube (Amicon Ultra 0.5 mL; Millipore Corp.) and centrifuged for 10 min at 13,500× *g*. Most glycopeptides were trapped on the 10 K ultra-filter, whereas most non-glycosylated peptides were filtered [15]. The trapped glycopeptide fraction was washed with 10 mM ammonium acetate in 10% (*v*/*v*) acetonitrile solution (400 μL), transferred to a 1.5-mL tube, and subjected to drying via vacuum centrifugation. Glycopeptides trapped on the filter were recovered and analyzed by UPLC-MS/MS.

© 2019 by the authors. Licensee MDPI, Basel, Switzerland. This article is an open access article distributed under the terms and conditions of the Creative Commons Attribution (CC BY) license (http://creativecommons.org/licenses/by/4.0/).
